# Effect of alpha-lipoic acid supplementation on the lipid profile and lipid ratios in women with gestational diabetes mellitus: A clinical trial study

**DOI:** 10.18502/ijrm.v18i12.8024

**Published:** 2020-12-21

**Authors:** Hadise Aslfalah, Mehri Jamilian, Hadi Ansarihadipour, Mahdi Abdollahi, Ali Khosrowbeygi

**Affiliations:** ^1^Students Research Committee, Arak University of Medical Sciences, Arak, Iran.; ^2^Department of Gynecology and Obstetrics, Endocrinology and Metabolism Research Center, School of Medicine, Arak University of Medical Sciences, Arak, Iran.; ^3^Department of Biochemistry and Genetics, Endocrinology and Metabolism Research Center, School of Medicine, Arak University of Medical Sciences, Arak, Iran.; ^4^Decorative and Hygienic Products, Control Laboratory of Food, Beverage, Food and Drug Administration, Arak University of Medical Sciences, Arak, Iran.; ^5^Traditional and Complementary Medicine Research Center (TCMRC), Arak University of Medical Sciences, Arak, Iran.

**Keywords:** Lipoic acid, Gestational diabetes, Lipids, Triglycerides, Cholesterol.

## Abstract

**Background:**

Evidence suggests that Oxidative stress has been shown to plays an important role in gestational diabetes mellitus (GDM) etiology. On the other hand, women with GDM are at an increased risk for complications such as endothelial dysfunction and cardiovascular diseases.

**Objective:**

To investigate the effects of alpha-lipoic acid (ALA) on the maternal circulating values of lipid profile and lipid ratios in women with GDM.

**Materials and Methods:**

Sixty women with GDM were participated in the present study. The ALA group (n = 30) received ALA (100 mg/day) and the placebo group (n = 30) received cellulose acetate (100 mg/day) for eight wk. The maternal circulating values of hemoglobin A1C, triglyceride (TG), total cholesterol, high-density lipoprotein cholesterol (HDL-C), low-density lipoprotein cholesterol, triglyceride-glucose (TyG) index, atherogenic index of plasma (AIP), non-HDL-C, and lipid ratios were assessed before and after the intervention. P-value < 0.05 was considered as statistically significant.

**Results:**

The values of TyG index (p < 0.001), TG (p = 0.006), TG/HDL-C (p = 0.003), and AIP (p = 0.005) decreased significantly in the ALA group after the intervention.

**Conclusion:**

Maternal circulating values of TyG index, TG, TG/HDL, AIP decreased after eight wk of ALA supplementation in women with GDM.

## 1. Introduction

Gestational diabetes mellitus (GDM) is a condition that is initiated or diagnosed for the first time in pregnancy, which is defined as glucose intolerance. This type of diabetes increases insulin resistance in body. Mothers suffering from GDM are at a high risk for type 2 diabetes in future. Clinical studies have shown that oxidative stress plays a role in the pathophysiology of numerous diseases (1). Normal pregnancy is regarded as a condition associated with increased oxidative stress. However, despite the increasing oxidative stress, there is a balance between antioxidants and oxidants in normal pregnancy. Bodies of evidence suggest that oxidative stress plays an important role in pregnancy complications such as GDM, preeclampsia, and hydatidiform mole (2, 3). Alpha-lipoic acid (ALA) (6, 8-thioctic acid or 1,2-dithiolane-3-pentanoic acid) is a sulfur-containing fatty acid (C8H14O2S2; MW 206.32) that plays a basic role as an antioxidant and is required for mitochondrial α-ketoacid dehydrogenase complex. ALA is produced by plants and animals. However, its synthesis in human is very low (4). Its antioxidant properties are attributed to several reasons including its capacity for direct scavenging of reactive oxygen species (ROS), regeneration of endogenous antioxidants such as glutathione, vitamins E and C, and its metal-chelating activity (5). Short-term treatment with ALA in patients with type 2 diabetes mellitus (T2DM) has been suggested to improve lipid profile by improving oxidative stress and inflammatory responses (6). A systematic review has shown that supplementation with ALA can improve lipid profile except HDL-C in patients with metabolic diseases (7).

A review study has suggested that ALA not only has no side effects but also has protective effects against embryopathy, fetal mortality, and placental ultrastructural changes due to diabetes during pregnancy (8). Women with a history of GDM are at an increased risk for complications such as endothelial dysfunction and cardiovascular diseases (9). It has been shown that lipid profile controlling during GDM can prevent impairment of the feto-placental endothelial function (10). On the other hand, women who use insulin during GDM can face some cardiovascular complications compared to those who undergo nutritional therapy for controlling the disease (11). However, little data are available about the effects of ALA supplementation on the values of lipid profile and lipid ratios in women with GDM. Therefore, the current clinical trial study was designed to investigate into this matter.

## 2. Materials and Methods

### Study registration and approval 

**Figure 1:**
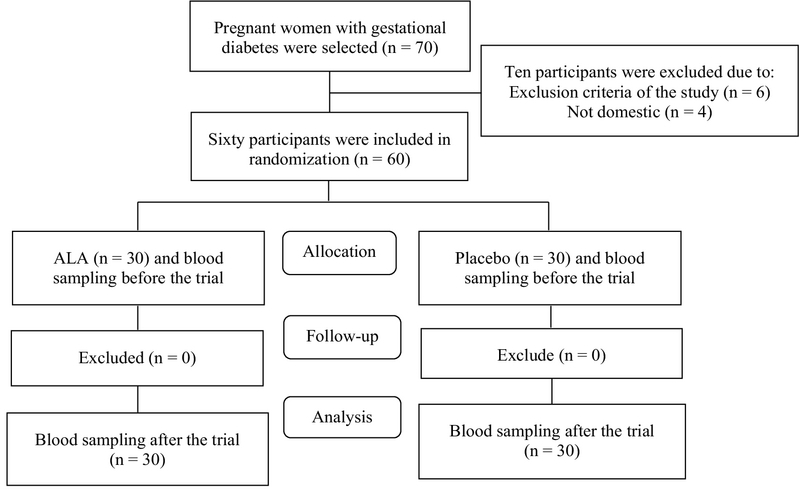
Consort diagram of ALA supplementation effects on lipid profile in women with gestational diabetes mellitus.

The current clinical trial study was conducted at the Taleghani Hospital and Diabetes Clinic of Arak University of Medical Sciences between February 2017 and January 2018. The study is a double-blind clinical trial in which the participants and the statistician were not aware of the type of drug to be administered and the group classification, respectively. GDM diagnosis was carried out according to the 75-gr oral glucose tolerance test when at least one or more of the following glucose values were exceeded: fasting ≥ 92 mg/dl; 1 h ≥ 180 mg/dL; and 2 h ≥ 153 mg/dL (12). The inclusion criteria of the study were: diagnosis of GDM, 18-40 yr of age, and being resident. The exclusion criteria, on the other hand, included: receiving antioxidant supplements at six-month intervals before the trial, preterm delivery, preeclampsia and other complications during the intervention stage, liver diseases, the need for insulin or medication during the intervention stage, absolute rest, smoking, and previous GDM history. Accordingly, 70 women, who were diagnosed with GDM by a specialist physician during 24 and 28 weeks of pregnancy, were selected using the convenience sampling method. Of them, 10 were excluded based on the inclusion criteria. Thus, 60 of them were enrolled in the current study (Figure 1).

### Randomization 

Participants were divided into ALA and placebo groups using simple randomization method by the random number table. While 30 participants were included in the ALA group and received ALA (Puritan's Pride, Oakdale, NY, USA) (1 capsule 100 mg/day), the remaining 30 were included in the placebo group and received manually prepared capsule containing cellulose acetate (Sigma Aldrich, St. Louis, USA) (1 capsule 100 mg/day). The intervention lasted for eight weeks. Studies have shown that a single dosage administration (50-600 mg) of ALA is completely absorbed within 30-60 min (8). Since receiving 100 mg/day of ALA has been shown to have no significant side effects on mothers and newborns during pregnancy (8), this dosage was used in the current study. All subjects were under the same diet plan for controlling blood glucose levels. Participants were asked to follow the provided diet plan and not to change their ordinary daily physical activity during the trial. As a reminder, every two weeks, the patients were phone called for taking their capsules appropriately.

### Assessment of variables 

Demographic data including participants' height, weight (light clothing without shoes weight), body mass index (BMI), blood pressure (right arm in sitting position) were measured at the beginning and end of the trial. BMI was determined as weight (kg)/height (m${}^{2}$). Venous blood samples were taken after 12 hr of fasting at the beginning and end of the intervention. Blood samples were centrifuged at 10,000 g for 15 min. Serum samples were stored at -80°C before analysis. To measure hemoglobin A1C (HbA1c), 1.5 ml of total blood was poured into EDTA-containing tubes that were assayed by chromatography kit (Biosystems, Barcelona, Spain).

Moreover, fasting blood sugar (FBS), high-density lipoprotein cholesterol (HDL-C), triglyceride (TG), and total cholesterol (TC) (ZiestChem Diagnostic, Tehran, Iran) were measured using a chemical analyzer (BT3500, Italy). Low-density lipoprotein cholesterol (LDL-C) levels were calculated using the Friedewald equation (13). The atherogenic index of plasma (AIP) (14), the triglyceride-glucose (TyG) index as Ln [(FBS × TG)/2] (15), the non-HDL-C, and the lipid ratios including TG/HDL-C, TC/HDL-C, LDL-C/HDL-C, and TG/TC were calculated (16). Levels of thiobarbituric acid-reactive substances (TBARS), a measure of lipid peroxidation, were determined for evaluating oxidative stress status (17). Serum ALA levels were measured by high-performance liquid chromatography method (KNAUER, Berlin, Germany) (18).

### Ethical consideration

The current study was approval by the Ethics Committee (IR.ARAKMU.REC.1395.343) of the Taleghani Hospital and Diabetes Clinic of Arak University of Medical Sciences.

### Statistical analysis 

Qualitative data were evaluated using the Chi-square test. The normal distribution of variables was explored by the Kolmogorov-Smirnov test. Comparisons of quantitative parameters were performed using paired and independent *t* tests. Interactions between the corresponding biochemical variables and the ALA group were explored using the Analysis of Covariance (ANCOVA). Data with p-values < 0.05 were considered as significant. All data are expressed as mean ± SEM. All statistical analyses were conducted using the SPSS, version 21 for windows (SPSS Inc., Chicago, IL, USA).

## 3. Results

In this study, data of the participants in two groups (n = 30/each) were analyzed. No significant differences were observed in the demographic and biochemical data of the two groups at the baseline (Tables I and II). In addition, no significant changes were observed in the weight, systolic blood pressure, and BMI in each group following the intervention.

Intragroup and intergroup comparisons of the variables are given in Tables III and IV, respectively. At the end of the intervention, values of FBS (p < 0.001), TyG index (p < 0.001), TG (p = 0.006), TG/HDL-C (p = 0.003), TG/TC (p = 0.004), AIP (p = 0.005), and TBARS (p < 0.001) significantly decreased in the ALA group compared with the baseline. Values of HbA1C decreased marginally in the ALA group (p = 0.059). No significant changes were observed in the values of TC (p = 0.189), LDL-C (p = 0.104), HDL-C (p = 0.328), non-HDL-C (p = 0.750), LDL-C/HDL-C (p = 0.727), and TC/HDL-C (p = 0.750) compared with the baseline in the ALA group. A comparison between the two groups showed a significant reduction in the serum levels of FBS (p < 0.001), TyG index (p = 0.022), TG (p = 0.041), TG/HDL-C (p = 0.016), TG/TC (p = 0.036), and AIP (p = 0.034) in the ALA group compared with the placebo group. However, no significant changes were observed in the values of HbA1C (p = 0.496), TC (p = 0.667), LDL-C (p = 0.441), HDL-C (p = 0.354), non-HDL-C (p = 0.978), LDL-C/HDL-C (p = 0.710), and TC/HDL-C (p = 0.357) between the two groups after the supplementation.

At the end of the trial, serum levels of ALA were increased in the ALA group (1.10 ± 0.10 vs 4.51 ± 0.24 µg/ml, p < 0.001), while no changes were observed in the placebo group (1.14 ± 0.07 vs 1.12 ± 0.07 µg/ml, p = 0.134).

Results of the ANCOVA test were significant for the TyG index (p = 0.001), TG (p < 0.001), TG/HDL-C (p = 0.006), AIP (p = 0.012), and TBARS (p = 0.007). However, it was not significant for the TG/TC (p = 0.308).

**Table 1:** Baseline biochemical characteristics of women with gestational diabetes mellitus in ALA and placebo groups


**Variables**	**ALA (n = 30)**	**Placebo (n = 30)**	**P-value**^¥^
**TG (mg/dl)**	250.69 ± 8.69	243.22 ± 9.01	0.553
**TyG index**	9.43 ± 0.04	9.38 ± 0.03	0.421
**TC (mg/dl)**	228.22 ± 3.99	234.70 ± 5.76	0.553
**HDL-C (mg/dl)**	43.99 ± 1.51	42.25 ± 1.04	0.347
**Non-HDL-C (mg/dl)**	184.23 ± 4.38	192.45 ± 5.87	0.266
**LDL-C (mg/dl)**	134.08 ± 4.64	143.80 ± 5.42	0.179
**TG/HDL-C**	5.86 ± 0.262	5.83 ± 0.238	0.938
**TC/HDL-C**	5.36 ± 0.213	5.66 ± 0.203	0.326
**LDL-C/HDL-C**	3.19 ± 0.189	3.49 ± 0.174	0.251
**AIP **	0.755 ± 0.02	0.756 ± 0.01	0.964
**TG/TC**	1.11 ± 0.04	1.04 ± 0.04	0.256
Note: Data are expressed as Mean ± SEM. ^¥^ Based on independent *t* test, TG: Triglyceride; TyG: Triglyceride-glucose; LDL-C: Low-density lipoprotein cholesterol; AIP: Atherogenic index of plasma; TC: Total cholesterol; HDL-C: High-density lipoprotein cholesterol			

**Table 2:** Baseline general characteristics of women with gestational diabetes mellitus in ALA and placebo groups


**Variables**	**ALA (n = 30)**	**Placebo (n = 30)**	**P-value**
Age (yr)*	30.96 ± 0.93	31.10 ± 0.92	0.918^¥^
Height (cm)*	163.00 ± 0.97	162.10 ± 1.21	0.579 ^¥^
Gestational age (wk)*	26.28 ± 0.23	26.51 ± 0.24	0.472^¥^
Family history of diabetes**	13 (43.3%)	12 (40.0%)	0.999${}^{\$}$
Weight (kg)*	70.70 ± 1.91	70.90 ± 2.04	0.942^¥^
BMI (kg/m${}^{2}$)*	26.64 ± 0.71	26.95 ± 0.73	0.793^¥^
SBP (mmHg)*	107.17 ± 2.49	108.00 ± 2.01	0.796^¥^
Note: * Data are expressed as Mean ± SEM. **Data presented as n (%), ^¥^Based on independent *t* test; ${}^{\$}$Based on Chi-square test BMI: Body mass index; SBP: Systolic blood pressure			

**Table 3:** Comparison of parameters within ALA and placebo groups before and after intervention in women with gestational diabetes mellitus


**Variables**	**ALA (n = 30)**			**Placebo (n = 30)**		
	**Before**	**After**	**P-value**	**Before**	**After**	**P-value**
HbA1C (%)*	5.29 ± 0.13	4.94 ± 0.13	0.059	5.31 ± 0.12	5.09 ± 0.16	0.274
FBS (mg/dl) *	101.43 ± 1.69	83.56 ± 1.31	<0.001${}^{‡}$	100.00 ± 1.24	94.63 ± 1.18	0.001${}^{‡}$
TG (mg/dl) *	250.69 ± 8.69	221.15 ± 6.91	0.006${}^{‡}$	243.22 ± 9.01	248.33 ± 11.01	0.611
TyG index*	9.43 ± 0.04	9.11 ± 0.04	<0.001${}^{‡}$	9.38 ± 0.03	9.33 ± 0.05	0.339
TC (mg/dl) *	228.22 ± 3.99	235.51 ± 4.32	0.189	234.70 ± 5.76	232.80 ± 4.53	0.739
HDL-C (mg/dl) *	43.99 ± 1.51	46.48 ± 1.81	0.328	42.25 ± 1.04	43.95 ± 1.99	0.463
Non-HDL-C (mg/dl) *	184.23 ± 4.38	189.03 ± 4.62	0.750	192.45 ± 5.87	188.84 ± 4.99	0.752
LDL-C (mg/dl) *	134.08 ± 4.64	144.80 ± 4.81	0.104	143.80 ± 5.42	139.17 ± 5.41	0.503
TG/HDL-C*	5.86 ± 0.262	4.91 ± 0.193	0.003${}^{‡}$	5.83 ± 0.238	5.87 ± 0.337	0.914
TC/HDL-C*	5.36 ± 0.213	5.27 ± 0.207	0.750	5.66 ± 0.203	5.57 ± 0.246	0.752
LDL-C/HDL-C*	3.19 ± 0.189	3.29 ± 0.184	0.727	3.49 ± 0.174	3.39 ± 0.215	0.703
AIP*	0.755 ± 0.02	0.680 ± 0.01	0.005${}^{‡}$	0.756 ± 0.01	0.748 ± 0.02	0.755
TG/TC*	1.11 ± 0.04	0.95 ± 0.03	0.004${}^{‡}$	1.04 ± 0.04	1.07 ± 0.05	0.567
Note: * Data are expressed as Mean ± SEM. ${}^{‡}$Significant at p < 0.05, based on paired *t* test HbA1C: Hemoglobin A1C; FBS: Fasting blood sugar; TG: Triglyceride; TyG: Triglyceride-glucose; LDL-C: Low-density lipoprotein cholesterol; AIP: Atherogenic index of plasma; TC: Total cholesterol; HDL-C: High-density lipoprotein cholesterol						

**Table 4:** Comparison of parameters between ALA and placebo groups before and after intervention in women with gestational diabetes mellitus


**Variables**	**ALA (n = 30)**		**Placebo (n = 30)**		**Pvalue**	
	**Before**	**After**	**Before**	**After**	**Before**	**After**
HbA1C (%)*	5.29 ± 0.13	4.94 ± 0.13	5.31 ± 0.12	5.09 ± 0.16	0.913	0.496
FBS (mg/dl) *	101.43 ± 1.69	83.56 ± 1.31	100.00 ± 1.24	94.63 ± 1.18	0.497	<0.001^¥^
TG (mg/dl) *	250.69 ± 8.69	221.15 ± 6.91	243.22 ± 9.01	248.33 ± 11.01	0.553	0.041^¥^
TyG index*	9.43 ± 0.04	9.11 ± 0.04	9.38 ± 0.03	9.33 ± 0.05	0.421	0.002^¥^
TC (mg/dl) *	228.22 ± 3.99	235.51 ± 4.32	234.70 ± 5.76	232.80 ± 4.53	0.553	0.667
HDL-C (mg/dl) *	43.99 ± 1.51	46.48 ± 1.81	42.25 ± 1.04	43.95 ± 1.99	0.347	0.354
Non-HDL-C (mg/dl) *	184.23 ± 4.38	189.03 ± 4.62	192.45 ± 5.87	188.84 ± 4.99	0.266	0.978
LDL-C (mg/dl) *	134.08 ± 4.64	144.80 ± 4.81	143.80 ± 5.42	139.17 ± 5.41	0.179	0.441
TG/HDL-C*	5.86 ± 0.262	4.91 ± 0.193	5.83 ± 0.238	5.87 ± 0.337	0.938	0.016^¥^
TC/HDL-C*	5.36 ± 0.213	5.27 ± 0.207	5.66 ± 0.203	5.57 ± 0.246	0.326	0.357
LDL-C/HDL-C*	3.19 ± 0.189	3.29 ± 0.184	3.49 ± 0.174	3.39 ± 0.215	0.251	0.710
AIP*	0.755 ± 0.02	0.680 ± 0.01	0.756 ± 0.01	0.748 ± 0.02	0.964	0.034^¥^
TG/TC*	1.11 ± 0.04	0.95 ± 0.03	1.04 ± 0.04	1.07 ± 0.05	0.256	0.036^¥^
weight (kg) *	70.70 ± 1.91	73.08 ± 1.79	70.90 ± 2.04	73.57 ± 2.13	0.943	0.863
Weight change (kg) *	2.38 ± 0.38		2.67 ± 0.55		0.676	
Note: *Data are expressed as Mean ± SEM. ^¥^` Significant at p < 0.05, based on independent *t* test. HbA1C: Hemoglobin A1C; FBS: Fasting blood sugar; TG: Triglyceride; TyG: Triglyceride-glucose; LDL-C: Low-density lipoprotein cholesterol; AIP: Atherogenic index of plasma; TC: Total cholesterol; HDL-C: High-density lipoprotein cholesterol						

## 4. Discussion

The current study showed that the maternal circulating values of TyG index, TG, TG/HDL-C, and AIP decreased after an eight-week supplementation with ALA in womenwith GDM. ALA supplementation at a dosage of 100 mg/day in women with GDM showed no significant changes in weight, systolic blood pressure, and BMI in both groups.

De Cicco and colleagues (19) found that the combination therapy with ALA and myo-inositol significantly reduces BMI in women with polycystic ovary syndrome. Noori and colleagues (20) found that consumption of 800 mg of ALA in diabetic nephropathy significantly decreases systolic blood pressure in the supplement group compared with the placebo group.

Orally consumed ALA is absorbed almost entirely from intestine (21). In the current study, values of ALA increased significantly in the treatment group compared with the placebo group after the intervention. Therefore, this finding can be accounted for ALA consumption by participants in the ALA group.

In addition, in the current study, HbA1C levels decreased marginally in the ALA group after supplementation with ALA, albeit the absence of statistical significance according to the results of ANCOVA. Accordingly, marginally reduced values of HbA1C may not be attributed to the intervention effects. Bao and co-workers (22) found that the daily consumption of 600 mg of ALA in patients with T2DM does not change HbA1C levels significantly. Chang and co-workers (23) observed that using 600 mg of ALA for 12 weeks does not affect HbA1c levels in individuals with end-stage renal disease. Porasuphatana and co-workers (24) observed that taking different doses of 300, 600, 900, and 1200 mg/day of ALA for six months causes a significant reduction in the HbA1C levels in individuals with T2DM. Derosa and co-workers (5) found that taking 600 mg of ALA supplementation in individuals with T2DM reduces HbA1C levels significantly.

An in vitro study conducted by Di Fulvio and co-workers (9) has shown that vascular cells obtained from umbilical cords of women with GDM maintain certain dysfunctions such as increased ROS and reduced nitric oxide (NO) bioavailability even after culturing for several times. Another in vitro study conducted by Di Tomo and colleagues (25) has shown that ALA significantly improves detrimental effects of hyperglycemia on umbilical vein endothelial cells obtained from mothers with GDM, concluding that this may point to new strategies for prevention of diabetes-related vascular complications.

A systematic review study has shown that supplementation with ALA can improve lipid profile except HDL-C in individuals with metabolic diseases (7). However, little data are available in this matter in GDM. In the current study, the mean TC/HDL and LDL/HDL level changes in the ALA and placebo groups did not show a statistically significant difference. However, the mean TyG index, TG, TG/TC, TG/HDL, and AIP levels in the ALA group significantly decreased after the intervention. It has been reported that the increased values of TyG index, TG, and TG/HDL-C in early pregnancy are associated with high incidence of GDM development (15). The TyG index is the best indicator of insulin resistance compared with other lipid parameters as a noninvasive, effective, and simple tool. On the other hand, TyG index is a better predictor of atherosclerosis than homeostatic model assessment of insulin resistance (HOMA-IR) (15, 26). AIP value has been shown to be a good biomarker for the risk of atherosclerosis and cardiovascular diseases. In addition, the calculation of AIP, especially when lipid profile is in normal range, has been proposed, which can show more useful data than traditional lipid analysis about patient's situation (14, 27). Results from the ANCOVA did not point to a statistically significant difference for TG/TC. Therefore, reduced TG/TC values may not be attributed to the intervention effects. An increase in the TG/HDL-C ratio was reported in GDM compared with healthy pregnancy (28). The TC/HDL-C, LDL-C/HDL-C, and TG/HDL ratios showed significant positive correlations with HOMA-IR. Therefore, these ratios could be used as simple alternative markers for assessing insulin resistance during pregnancy and predicting women at a high risk for GDM (29). It has been shown that ALA can increase adenosine monophosphate-activated protein kinase (AMPK) activity which causes inhibition of fatty acid synthesis, while simultaneously fatty acids oxidation is activated. On the other hand, expression of enzymes acetyl-CoA carboxylase and fatty acid synthase, two rate-limiting enzymes in fatty acid synthesis, are depressed in subjects supplemented with ALA (7).

A major limitation of the present study was the lack of a healthy control group to allow the comparison of the results obtained from the participants with them.

## 5. Conclusion

The results of the current study in women with GDM showed a significant decrease in the maternal circulating values of TyG index, TG, TG/HDL, AIP, and TBARS in the ALA group compared to the placebo group at the end of the intervention. More studies are required to evaluate the potential effects of ALA on the reduction of birth defects in women suffering from GDM.

##  Conflict of Interest

The authors declare that they have no conflict of interest.
